# Impact of Hearing Aid Amplification on Subjective Tonal Tinnitus in Patients with Gently Sloping and Ski-Slope Hearing Loss: A Retrospective Cohort Study

**DOI:** 10.3390/audiolres15060167

**Published:** 2025-12-03

**Authors:** Daniele Portelli, Sabrina Loteta, Cosimo Galletti, Mariangela D’Angelo, Leonard Freni, Pietro Salvago, Francesco Ciodaro, Giuseppe Alberti

**Affiliations:** 1 Unit of Otorhinolaryngology, Department of Adult and Development Age Human Pathology “Gaetano Barresi”, University of Messina, 98125 Messina, Italy; daniele.portelli09@gmail.com (D.P.); mariangeladangelo17@gmail.com (M.D.); leonardfreni@gmail.com (L.F.); dottfciodaro@alice.it (F.C.); galberti@unime.it (G.A.); 2 Faculty of Medicine and Surgery, Kore University of Enna, Piazza dell’Università, 94100 Enna, Italy; cosimo.galletti01@unikore.it; 3 Dipartimento di Biomedicina, Neuroscienze e Diagnostica Avanzata (BiND), Sezione di Audiologia, Univesità degli Studi di Palermo, Via del Vespro 129, 90127 Palermo, Italy

**Keywords:** tinnitus, tinnitus treatment, hearing aid, hearing loss, ski-slope

## Abstract

**Background/Objectives**: This study aims to evaluate the effectiveness of hearing aid amplification in reducing self-perceived tinnitus handicap in individuals with ski-slope hearing loss—a population seldom addressed in previous research. In addition, a correlation analysis was performed to examine the relationship between tinnitus duration, pitch, loudness, and THI scores. The results are then compared with those of patients with high-frequency gently sloping hearing loss. **Methods**: 38 patients with bilateral sensorineural hearing loss and chronic tonal tinnitus were retrospectively evaluated and divided into two equal groups: high-frequency gently sloping and ski-slope hearing loss (n = 19 each). Tinnitus pitch, loudness, and edge frequency were assessed. The Mann–Whitney test compared tinnitus characteristics between groups, while the Wilcoxon signed-rank test evaluated pre- and post-treatment THI scores. Spearman correlation was used to explore associations between tinnitus duration, intensity, pitch, and THI outcomes. **Results**: The Mann–Whitney test showed significant differences in tinnitus pitch, and edge frequency between both groups; no statistically significant differences were found for the tinnitus level. Tinnitus frequency was higher in the high-frequency gently sloping group. The Wilcoxon test confirmed significant improvements in THI scores post-treatment for both groups (*p* < 0.001). No significant correlations were found between tinnitus duration, level, pitch, and post-treatment THI scores. **Conclusions**: Hearing aids effectively reduce tinnitus severity in patients with ski-slope and gently sloping hearing loss, supporting their use as a therapeutic option. Larger, multicentric studies are recommended to validate these findings and explore specific auditory profiles and processing strategies.

## 1. Introduction

Tinnitus is a common, debilitating and life-changing symptom. It is an auditory sensation perceived by an individual in the absence of an external sound source [1].

Tinnitus is experienced by approximately 8–30% of the adult population, with prevalence increasing with age [2]. It affects both males and females equally, while in children it is challenging to ascertain the presence of tinnitus since they tend to tolerate the symptom better [1,2]. Conversely, it can also be constant or intermittent. Tinnitus is associated with various risk factors, including otological (otitis media, labyrinthitis, vestibular schwannoma, sensorineural hearing loss, Ménière’s disease, otosclerosis, presbycusis, noise exposure), neurological (meningitis, migraine, multiple sclerosis, epilepsy), traumatic (head or neck injury), cardiovascular (hypertension), rheumatological (rheumatoid arthritis), immunological (systemic lupus erythematosus, systemic sclerosis), endocrine-metabolic (diabetes mellitus, hyperinsulinemia, hypothyroidism), and pharmacological, particularly in the case of ototoxic drugs (analgesics, antibiotics, antineoplastic drugs), etc. [1,2,3]. One or both ears may be affected, and in the latter case, patients often perceive the tinnitus as centred in their head [1].

Subjective tinnitus is commonly associated with otological conditions, particularly high-frequency hearing loss, and is thought to result from neuroplastic responses to sensory deprivation [3,4,5]. Although cochlear damage may initiate tinnitus, the persistent perception of sound is maintained by neural changes in the central auditory system.

Pathophysiologically, subjective tinnitus may stem from abnormal spontaneous activity or synchronization of neural firing in the auditory system [4,5].

Three main models have been proposed to explain tinnitus pathophysiology: the tonotopic reorganization model, the neural synchrony model, and the “hidden hearing loss” hypothesis [6].

According to the tonotopic reorganization model, hearing loss leads to an altered tonotopic arrangement in the primary auditory cortex [4]. Neurons in the affected cochlear region adopt tuning properties of neighbouring functioning areas, creating a correlation between the audiogram and tinnitus pitch [4]. The boundary between normal hearing and hearing loss in the audiogram called the “edge frequency,” is often associated with the tinnitus pitch. Supporting this, König et al. confirm a link between tinnitus pitch and edge frequency, while Moore et al. observe a stronger match with the lower edge frequency [7,8].

In the neural synchrony model, tinnitus arises from increased neural synchronisation within the region of impaired hearing. According to this hypothesis, the tinnitus pitch aligns with the frequency range of the affected hearing region [9,10].

In approximately 80–90% of cases, tinnitus is typically associated with varying degrees of hearing loss, although the degree of hearing impairment is not a prognostic factor for the tinnitus itself.

The severity of subjective tinnitus varies widely, with some individuals experiencing minor disruptions and others facing significant distress that can impact their emotional well-being and quality of life. Therapeutic approaches for subjective tinnitus are diverse, as there is no single treatment that universally alleviates symptoms.

Due to the complexity of the neural and pathophysiological mechanisms involved in tinnitus generation, a variety of treatment options have been proposed over the years. Several approaches have been explored, yielding mixed results in terms of symptom improvement. These include pharmacological treatments, cognitive behavioural therapy (CBT), tinnitus retraining therapy (TRT), cochlear implants, bio and neurofeedback, invasive brain stimulation, non-invasive brain stimulation, sound therapy and hearing aids use [1,11,12].

The goal of active sound stimulation approaches, such as tailor-made notch music training, frequency discrimination training, and auditory perceptual training, or traditional passive stimulation like wideband noise generators (maskers), hearing aids, or a combination of these approaches, is to provide relief from tinnitus perception [12].

Sound therapy can also be provided through hearing aids. According to the European guidelines (2019), there is a weak recommendation for using hearing aids in the treatment of tinnitus, as existing literature is based on low-quality studies [11,13,14]. However, in a recent literature review analysing 34 studies, Kikidis et al. (2021) concluded that simply fitting a hearing aid in patients with subjective tinnitus associated with hearing loss can be considered an effective management and treatment strategy for tinnitus [11]. We, the authors, believe that the use of more advanced digital hearing aids, equipped with specialised technologies and sophisticated sound processing strategies, could have a greater impact on managing tinnitus in patients with associated hearing loss compared with simple acoustic amplification alone [15,16,17]. The aforementioned tinnitus pathophysiology models could provide a framework to understand how hearing aid amplification may affect tinnitus perception. Tonotopic reorganisation and neural synchrony models would suggest that restoring auditory input through amplification could help reduce maladaptive cortical changes and aberrant activity, whereas the “hidden hearing loss” hypothesis posits that tinnitus may result from cochlear synaptopathy even when audiometric thresholds appear normal, potentially making it less responsive to the various treatment [5,6].

A recent double-blind, randomised clinical trial demonstrated that hearing aids can lead to long-term tinnitus suppression, regardless of the use of frequency-lowering algorithms [17]. The scientific literature contains numerous studies evaluating the benefits of hearing aids on tinnitus perception, predominantly focusing on patients with sloping sensorineural hearing loss.

High-frequency gently sloping hearing loss was defined as an audiometric curve with a dB HL gap between consecutive frequencies (125, 250, 500, 700, 1000, 1500, 2000, 3000, 4000, 6000, 8000) of 30 dB HL or less. For ski-slope hearing loss, the criteria defined by Schuurbiers et al. (2017) were used, specifically, hearing thresholds of 25 dB or less at 250 Hz, 80 dB or more at 4 kHz, and a decline of 70 dB or more between 500 Hz and 4 kHz or 40 dB or more between 250 Hz and 1 kHz or between 500 Hz and 2 kHz [18].

What sets our work apart is its investigation into whether hearing aids can effectively mitigate the impact of tinnitus-related handicap in this specific patient group, an area that remains underexplored in the existing literature.

In the recent literature review by Kikidis et al. (2021), several studies were analysed to assess the effects of hearing aid amplification in patients with tinnitus [11,19]. Notably, it was highlighted that only one of these studies specifically included patients with ski-slope hearing loss. This observation forms the rationale behind our investigation, aiming to explore this underrepresented patient subgroup. The present study seeks to evaluate the effectiveness of hearing aid amplification in reducing self-perceived tinnitus handicap in individuals with ski-slope hearing loss—a population seldom addressed in previous research. In addition, a correlation analysis was performed to examine the relationship between tinnitus duration, pitch, loudness, and THI scores.

The underlying hypothesis of our study is that patients with ski-slope hearing loss may derive less benefit from hearing aid amplification, as they often have limited residual hearing at high frequencies, which are probably essential for the masking of tinnitus.

## 2. Materials and Methods

### 2.1. Participants

The study was conducted at the University of Messina, within the ENT Unit at “G. Martino” Polyclinic in Messina. Data from 38 patients, consisting of 13 females (34,2%) and 25 males (65,8%) were retrospectively analysed.

Inclusion criteria for the study included patients with bilateral sensorineural hearing loss eligible for hearing aid use, bilateral subjective chronic tonal tinnitus with no specific underlying cause (such as nervous system tumours or neurological diseases), patients with no prior hearing aid use, and adults aged 18 years or older. “Tonal tinnitus” is defined as a type of tinnitus characterised by the perception of a single, continuous tone, as described by Moore et al. (2010) [7].

**Figure 1:**
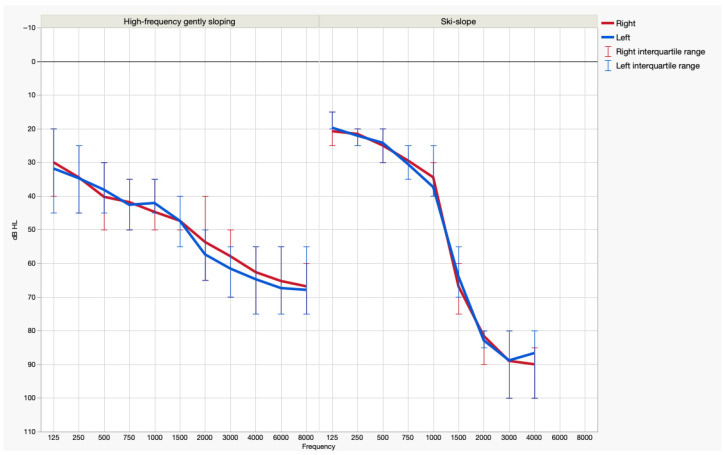
Patients’ air conduction hearing thresholds (means and interquartile ranges).

The patients were divided into two groups based on hearing loss morphology: high-frequency gently sloping hearing loss (n = 19, 9 females, 10 males) and ski-slope hearing loss (n = 19, 4 females, 15 males) (Figure 1).

The study complies with the Declaration of Helsinki. During the counselling, all participants provided consent for hearing aid application, audiological testing, and the use of their data. Local ethical committee approval Prot. 97-24, 19 July 2024.

### 2.2. Subjective Scales

The Tinnitus Handicap Inventory (THI) is a widely used assessment questionnaire designed to evaluate the impact of tinnitus on a patient’s daily life [20]. It consists of 25 questions across three subscales: functional, emotional, and catastrophic. These questions address the effect of tinnitus on concentration, sleep, emotional well-being, and social interactions.

The THI scoring system assigns points based on patients’ responses to 25 questions. Each question offers three response options: “yes” (worth 4 points), “sometimes” (2 points), and “no” (0 points). The total score, which ranges from 0 to 100, is calculated by summing the points for all questions. The score reflects tinnitus severity, classified into five levels: slight (0–16), mild (18–36), moderate (38–56), severe (58–76), and catastrophic (78–100) [20]. In this study, the THI was used as the primary assessment tool for evaluating the changes in self-perceived tinnitus handicap.

### 2.3. Hearing Aids

The Widex Moment RIC-312 220, 330, and 440 models were used as hearing aids for both groups in this study, all of which fall into the Receiver-In-Canal (RIC) category. Receiver power types, available in M, P, and HP types, were chosen based on the individual’s audiometric profile and fitting software recommendations. The coupling system—open dome, tulip dome, or closed dome—was also selected in line with software suggestions to ensure that target amplification levels set by the prescriptive method were achieved. The chosen prescription method was the NAL-NL2. This decision was made with consideration of Kikidis et al. (2021), who in their literature review noted that most studies reporting on prescriptive formulas used NAL (NL1 and NL2) [11]. For this reason, we prefer to use the NAL-NL2 prescriptive method for patients with bilateral hearing loss and tinnitus, as it aligns with most of the available research evidence and allows for consistent target amplification in this population.

Decisions on hearing aid type were made in collaboration with the patient, considering factors such as hearing loss, cost, aesthetics, ease of use, and maintenance needs. Audiogram data were uploaded into the fitting software (Widex Compass GPS V.4) to generate the target amplification curve. The initial setup included in situ pure tone audiometry (Sensogram) and anti-feedback calibration. The hearing aid was configured with full noise reduction and listening optimization features for an optimal listening experience.

In subjects with ski-slope hearing loss, the “Audibility Extender” algorithm by Widex was enabled. This feature uses Linear Frequency Transposition, with the parameters of “transposed sound volume” and “start frequency” set according to recommendations from the fitting software, based on the algorithm’s calculation of the patient’s audiometric thresholds [21,22].

During the first four weeks, patients were seen weekly by an audiologist, who performed gradual amplification adjustments to facilitate acclimatisation to the device.

### 2.4. Audiological Tests and Procedure

The standard hearing aid fitting protocol included an initial phase with otomicroscopic and tympanometric examinations to rule out acute, external or middle ear manageable pathologies. Following this, pure tone and speech audiometry were conducted to determine candidacy for hearing aid use. Pure-tone and speech audiometry were conducted in a soundproof booth using TDH39 headphones and the B71 bone vibrator.

The Pure Tone Average (PTA) was used in the analysis, calculated as the average of air conduction thresholds at the central frequencies of 500, 1000, and 2000 Hz.

The edge frequency was calculated following the procedure described by Moore et al. (2010). We define the “edge frequency” as the boundary between a region of normal or near-normal hearing and a region with greater hearing loss. The same procedure outlined by Moore et al. was carried out as follows [7]:

Audiometric thresholds were measured at frequencies 125, 250, 500, 750, 1000, 1500, 2000, 3000, 4000, 6000, and 8000 Hz, designated as F1, F2, F3, F4, F5, F6, F7, F8, F9, F10, and F11, respectively.

The threshold difference between two successive frequencies was calculated (e.g., F2-F1, F3-F2, etc.).

When one difference (Δn) was larger than all other Δn values, the edge frequency was identified as the lower of the two frequencies for which Δn was the largest. If there were two equal largest values of Δn, and these two were adjacent to one another in frequency, then the lower one was used. If there were two equal largest values of Δn, and these two were not adjacent to one another in frequency, then it was assumed that there were two edge frequencies, each corresponding to the lower of the two frequencies for which Δn was the largest [7].

The subject was asked to compare the pitch and level of their tinnitus with that of a sinusoidal tone delivered via the audiometer. Several steps were performed to match the pitch and level of the presented tone as closely as possible to the patient’s perceived tinnitus. This matching process was conducted separately for each ear. The tone was presented ipsilaterally to the ear with the tinnitus to be matched. Initially, frequencies from the audiometric thresholds (125, 250, 500, 750, 1000, 1500, 2000, 3000, 4000, 6000, and 8000 Hz) were tested, and the tone was delivered at 5 dB above the highest recorded audiometric threshold for each frequency. The patient was then asked to identify which frequency most closely matched the pitch of their tinnitus.

Subsequently, the tone frequency was adjusted up or down by increments of 100 Hz based on the patient’s feedback to further refine the match. Once the patient identified a frequency like their tinnitus, the tone was presented at the matched frequency, an octave above, and an octave below, to avoid octave errors, and the patient indicated the closest match. For patients with ski-slope hearing loss, testing one octave above the tinnitus frequency was not always feasible, as there were often no residual hearing thresholds in those higher frequencies. In such cases, only the lower octave was tested. After identifying the pitch, a similar procedure was applied for level matching, adjusting the pure tone in steps of 5 dB. In patients with ski-slope hearing loss, the procedure was performed exclusively at frequencies where the patient was able to respond to the auditory stimulus.

To minimize the confounding effect of tinnitus perception in the contralateral ear during testing, pink noise at 10 dB SL (10 dB above the threshold PTA) was delivered via air conduction to the non-tested ear. The decision to use contralateral masking was made by the authors to mitigate the potential impact of tinnitus perception in the contralateral ear during testing. Tinnitus can be masked using both pure tones and noise stimuli [23]. Prolonged exposure to these sounds may temporarily reduce or suppress tinnitus, not only during the presentation of the stimulus but also shortly after it stops [23]. Tinnitus in one ear can sometimes affect the ability to accurately perceive or identify auditory stimuli in the opposite ear, particularly when the tinnitus is loud or intrusive. By delivering a contralateral masking sound at a level of 10 dB HL above the PTA in the non-tested ear, we aimed to reduce the distraction from the side contralateral to the tested ear and improve the accuracy of the pitch matching process [23]. This approach helps ensure that the tinnitus in the ear under test was the primary focus of the auditory matching task, without interference from the tinnitus in the opposite ear.

It is essential to clarify that this study does not aim to pinpoint the exact tinnitus frequency meticulously. The matching process was carried out approximately due to two main reasons: the time required for a precise matching procedure can be lengthy, potentially impacting patient compliance, and in our hearing aid fitting setting, the primary interest is determining whether the tinnitus falls within the amplification band provided by the hearing aid to utilize the potential masking effect on the tinnitus itself. For these reasons, the procedure was carried out using 5 dB steps, also taking into account potential clinical applicability and the need for a more rapid execution.

The use of dB HL rather than dB SL (Sound Level) was chosen to ensure consistency with standard clinical audiometric reporting and to facilitate comparison across patients with different hearing thresholds. Since dB HL values are referenced to normative threshold values and are routinely used in audiological evaluations and hearing aid fittings, they provide a standardised and interpretable frame of reference. Furthermore, because the purpose of our analysis was not to assess suprathreshold perception per se, but rather to document audiometric thresholds and presentation levels in a clinically applicable way, dB HL was deemed more appropriate for the context of this study.

All tests were executed using the Madsen Astera2 audiometer (Otosuite V. 8.84.0 software).

Patients presented to our centre either for hearing loss or tinnitus, and in cases where the hearing loss was deemed suitable for amplification, the use of a hearing aid was proposed. Once eligibility for hearing aid use was confirmed, the device was discussed with the patient, and a suitable model was chosen based on individual hearing loss characteristics and personal preferences. During this same phase, all patients received counselling. The counselling provided in this study comprised educating patients about the nature of tinnitus, including its pathophysiology and benign character, clarifying that it represents a symptom rather than a disease. It also included setting realistic expectations, emphasising that while complete elimination of tinnitus is rarely achievable, interventions can reduce its impact and improve quality of life. Furthermore, patients were offered practical coping strategies, such as avoiding complete silence, using background noise, and adopting relaxation techniques to mitigate stress. Counselling also addressed sleep hygiene, encouraging regular sleep schedules and the avoidance of stimulating activities before bedtime, given that poor sleep may exacerbate tinnitus perception. Importantly, none of the patients underwent any other form of tinnitus therapy during the study period.

For patients with tinnitus, our protocol also included determining the edge frequency, level, and pitch of the tinnitus according to the methods outlined above. Additionally, the THI questionnaire was administered to these patients to assess the tinnitus-related handicap baseline.

The hearing aid fitting process then began, with insertion gain gradually increased over approximately four weeks. Patients were seen weekly during this period for gain adjustments. After about four weeks, real ear measurements (REMs) were conducted to verify if the prescriptive target gain, as indicated by the selected prescription method, has been achieved. Any discrepancies between the prescribed and real gain were adjusted at this stage. Patients were instructed to report any problems related to sound quality so that these could be addressed during the preliminary phase and at the time of the REMs.

Patients were instructed to wear their hearing aids continuously throughout the day, allowing for removal only in situations involving potential water exposure or during nighttime hours. Data on hearing aid usage time were obtained through datalogging, available within the hearing aid fitting software. Patients who did not achieve an average daily use of at least 15 hours were not included in this retrospective study, in order to ensure that the reported outcomes reflected the effect of consistent and effective hearing aid use. Six months after the REMs, the THI questionnaire was re-administered to assess the impact of the “effective” hearing aid amplification on tinnitus distress. For this study, no adjustments were made to either the acoustic amplification or the coupling system during the six months. Patients were seen once a month solely to ensure that no technical issues were affecting the functioning of the hearing aids. The choice of a six months was based on the intention to allow sufficient time for both the auditory and cognitive systems to adapt to the new condition of acoustic amplification.

### 2.5. Statistical Analysis

The numerical data were expressed as the median and interquartile range (Q1–Q3), and the categorical variables as absolute frequencies and percentages.

The Kolmogorov–Smirnov test was used to assess the normality of the numerical variables; however, as these were not normally distributed and due to the small sample size, a non-parametric approach was employed.

The Mann–Whitney test was applied to compare tinnitus duration, PTA, edge frequency, tinnitus intensity, tinnitus frequency, pre- and post-treatment THI between patients with high-frequency gently sloping hearing loss and those with ski-slope hearing loss.

The non-parametric Wilcoxon signed-rank test for paired samples was used to compare pre-treatment and post-treatment Tinnitus Handicap Inventory (THI) scores in both groups (high-frequency gently sloping vs. ski-slope hearing loss). A boxplot was created to better visualize the THI results.

The Spearman correlation test was applied to assess the possible correlation between tinnitus duration and level, tinnitus duration and post-treatment THI, tinnitus pitch and post-treatment THI.

Additionally, a descriptive analysis of THI values in terms of the minimal clinically important difference (MCID) was performed between the two groups, following the study by Engelke et al. (2025). In this analysis, a MCID of 11-point reduction in the THI was considered as the threshold for clinically meaningful improvement [24].

Statistical analyses were performed using SPSS 27.0 for the Windows package. A *p*-value lower than 0.05 was statistically significant and was reported in bold in the text and the table.

## 3. Results

### 3.1. Demographics

The sample consists of 38 patients (65.8% male and 34.2% female) (Table 1). The median tinnitus duration was 4 years for both groups.

The median values and the interquartile range (Q1–Q3) for pitch, level, and edge frequency are shown in Table 2.

### 3.2. Tinnitus

The Mann–Whitney test revealed statistically significant differences in the tinnitus pitch, and the edge frequency for either the right or left ear and the pre-treatment THI in both groups; no statistically significant differences were found for the tinnitus level for the right and left ear between groups (Table 2).

The tinnitus frequency was found to be higher and statistically different in both ears for the high-frequency gently sloping group compared to the ski-slope group. Conversely, tinnitus intensity, for both the right and left ear, was not statistically significant.

### 3.3. THI Changes

**Figure 2:**
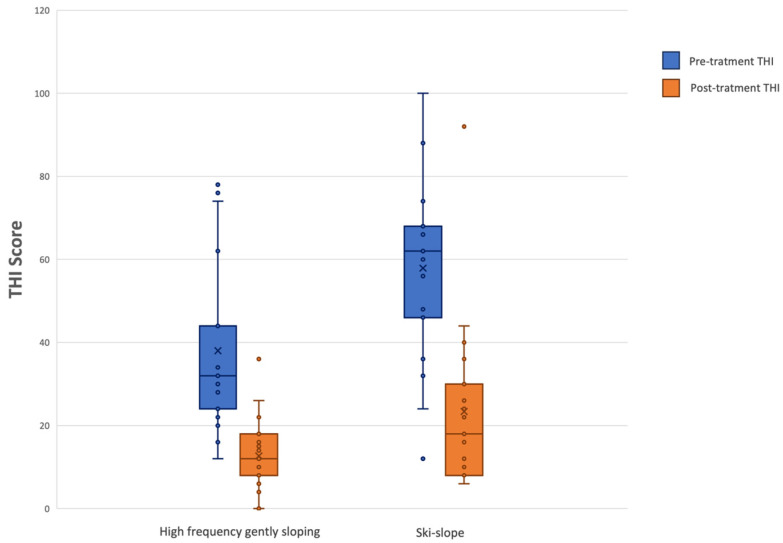
Boxplot representing THI scores.

The non-parametric Wilcoxon signed-rank test for paired samples was used to compare pre-treatment and post-treatment THI scores (after 6 months) between patients with high-frequency gently sloping hearing loss and those with ski-slope hearing loss. A statistically significant difference was identified in both groups, with a *p*-value of < 0.001 for both high-frequency gently sloping and ski-slope hearing loss groups (Figure 2, Table 3).

In terms of MCID, a reduction greater than 11-points was observed in 16 out of 19 cases for both the high-frequency gently sloping and ski-slope groups. Specifically, in the high-frequency gently sloping group, the distribution of THI score improvements ranged from 14 points at the 25th percentile, to 24 points at the median, and 29 points at the 75th percentile. In the ski-slope group, the corresponding values were 24, 36, and 48 points, respectively. These results indicate a greater reduction in THI scores for the ski-slope group compared to the high-frequency gently sloping group.

### 3.4. Correlations

**Figure 3:**
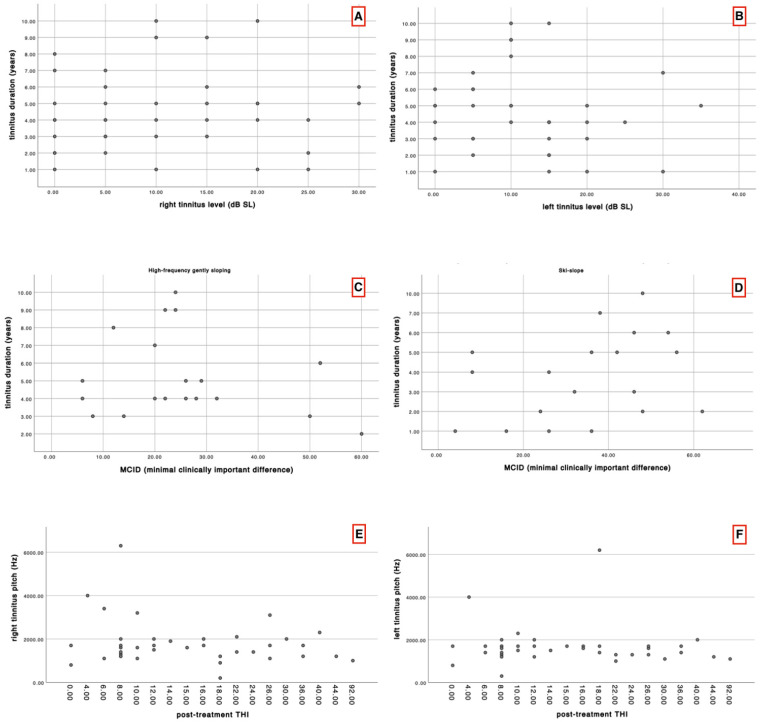
Scatterplots of correlation analysis. (**A**) scatterplot showing the correlation between tinnitus duration and right tinnitus level; (**B**) scatterplot showing the correlation between tinnitus duration and left tinnitus level; (**C**) scatterplot showing the correlation between tinnitus duration and the MCID for the high frequency gently sloping group; (**D**) scatterplot showing the correlation between tinnitus duration and the MCID for the ski-slope group; (**E**) scatterplot showing the correlation between right tinnitus pitch and post-treatment THI; (**F**) scatterplot showing the correlation between left tinnitus pitch and post-treatment THI.

Additionally, using the tinnitus duration and tinnitus level data, we aimed to investigate whether a correlation existed between these two variables. For this purpose, Spearman’s correlation test was applied, which did not reveal any significant correlation (Table 4, Figure 3A,B).

Using the same test, we evaluated whether there was a correlation between tinnitus duration and the treatment efficacy to determine if tinnitus duration could influence the benefit gained from acoustic stimulation. In this regard, a bivariate correlation was performed for each group separately, comparing tinnitus duration with the MCID, without, however, revealing significant correlations in either group (Table 5, Figure 3C,D).

Similarly, tinnitus pitch was correlated with post-treatment THI scores (Table 6, Figure 3E,F). However, these tests also proved a non-statistically significant correlation.

## 4. Discussion

The present study demonstrated statistically significant differences between the two groups in terms of tinnitus pitch, and edge frequency. Despite these perceptual differences, both groups experienced statistically significant improvements in THI scores following six months of hearing aid use. This supports the notion that amplification may be beneficial in reducing tinnitus-related handicap, irrespective of audiogram morphology, although the mechanisms by which this occurs may differ between groups. Tinnitus duration was not correlated with tinnitus level, and tinnitus pitch showed no correlation with post-treatment THI scores. Likewise, the MCID was not significantly correlated with tinnitus duration in either group.

Based on our initial hypothesis that tinnitus perceptual characteristics or the audiometric profile might influence the benefit from hearing aid use, we expected that differences in tinnitus pitch or edge frequency could lead to varying improvements in THI scores. However, our results indicate that, despite significant differences between groups in pitch and edge frequency, these characteristics do not affect THI improvement. Both groups experienced comparable and significant reductions in THI scores after six months of amplification, suggesting that hearing aid use can reduce tinnitus-related handicap regardless of tinnitus perceptual features or audiogram morphology.

Indeed, according to the theory of cortical reorganization, the therapeutic effect of acoustic stimulation may result from sound enrichment across the frequencies affected by hearing loss, which helps alleviate the neighbouring over-activated region (corresponding to the tinnitus pitch). For instance, if a continuous masking sound presented above the threshold is played to the patient for 30–60 seconds and then switched off, the tinnitus sensation often vanishes temporarily, and reappears after a few seconds or minutes [4].

According to Kikidis et al. (2021) in their literature review found that hearing aids improve tinnitus by up to 50% in 40% to 85% of patients reviewed [11]. This improvement, associated with an improvement in auditory outcomes, appears to be independent of the specific hearing aid used [16]. However, the authors note that different prescriptive fitting formulas, which provide varied gain patterns, might influence these outcomes [11,15].

Jalilvand et al. (2015) compared the effects of hearing aids and noise generators on chronic tinnitus treatment, finding that hearing aids provided better outcomes after six months [25]. While noise generators offered early-stage benefits, patients found them challenging long-term as they masked both tinnitus and essential environmental and speech sounds. Consequently, patients preferred hearing aids, as they not only suppressed tinnitus perception but also enhanced speech understanding and environmental sound perception by simultaneously addressing underlying hearing loss [25]. Additionally, both hearing aid amplification alone and in combination with noise generators have proven equally effective in reducing tinnitus discomfort in patients with mild to moderate hearing loss [26].

In our study, we aimed to evaluate whether acoustic stimulation provided by hearing aids could be effective for patients with both ski-slope hearing loss and tinnitus.

Regarding this aspect, we, the authors, find it crucial to emphasize an important point concerning tinnitus. Tinnitus loudness did not differ significantly between the two groups, suggesting that loudness may not be a distinguishing feature across different audiometric configurations. Conversely, tinnitus pitch and edge frequency were significantly different, indicating that these parameters are more strongly linked to the specific morphology of hearing loss. The observed difference in edge frequency may be attributed to the distinct audiometric profiles associated with the two types of hearing loss. Moreover, pre-treatment THI scores were found to be statistically significant between groups, with higher scores observed in patients with ski-slope hearing loss.

These findings suggest a distinct tinnitus perceptual profile associated with different audiometric configurations, potentially reflecting underlying variations in cochlear damage or neural plasticity. It should also be considered that unaided sound deprivation may contribute to increased overall stress, potentially exacerbating tinnitus perception and handicap. The mechanisms driving tinnitus are complex and not yet fully understood. Therefore, our study also aims to investigate tinnitus characteristics of the different audiometric curve morphologies.

Furthermore, it is well established that a hearing loss exceeding 70 dB HL is commonly associated with cochlear dead regions [27,28].

In the patients we analysed, the tinnitus pitch falls within the frequency range amplified by the hearing aid. This amplification likely produces a masking effect on the tinnitus, thereby providing relief. However, a limitation of assessing tinnitus in patients with ski-slope hearing loss is that the tinnitus pitch may correspond to a higher frequency than reported, potentially in regions of the audiogram where the individual has no residual hearing. In such cases, accurately estimating the tinnitus pitch becomes challenging, especially since these patients typically exhibit similar hearing loss patterns in both ears. From a purely clinical and practical standpoint, we believe that accurately estimating the tinnitus frequency, intensity, and consequently the edge frequency is particularly important in patients with ski-slope hearing loss, to optimise hearing aid fitting. It should also be considered that these patients often require the application of frequency-lowering algorithms, which may influence tinnitus perception and potentially alter the efficacy of acoustic stimulation.

Moreover, a key element of the hearing aid fitting process is the ongoing interaction between patient and audiologist. This relationship supports the fine-tuning of hearing aid settings and ensures patients are properly educated on their use. Regular follow-up visits allow for adjustments based on patient feedback and create a supportive setting to address concerns and offer counselling. This continued guidance promotes adaptation to amplification and has been shown to improve treatment outcomes [29,30].

Regarding the pre- and post-hearing aid fitting THI scores, a statistically significant difference was observed between pre- and post-treatment THI scores for both groups. This supports the notion that amplification may be beneficial in reducing tinnitus-related handicap, irrespective of audiogram morphology, although the mechanisms by which this occurs may differ between groups. However, it is important to note that the benefit could be closely related to the masking effect that amplification has on tinnitus, which may make it less effective in quiet environments. Nevertheless, by enriching the overall soundscape through the amplification of ambient sounds, hearing aids may still contribute to making tinnitus more tolerable even under these conditions. This highlights the importance of using hearing aids for as long as possible throughout the day. Additionally, even in quiet conditions, there are always background environmental noises that are amplified (even partially) which help recreate an adequate acoustic scene that is often distorted by patients with hearing loss. We believe that the amplification of these sounds could be the reason why tinnitus is better tolerated in these situations when the hearing aids are worn. In a study by Tyler et al. (2012), the authors concluded that for some patients, forms of total or partial low-level masking could provide relief [31]; notably, the patients considered in that clinical trial were not hearing aid users [31]. Conversely, in another study by the same author, partial masking was found to be beneficial [32]. Finally, in a later study by Tyler et al. (2021), comparing hearing aid users and non-users, it was reported that some patients experienced benefit whereas others did not, with highly variable results [33]. It is important to emphasise that our study did not employ dedicated masking sounds; rather, the probable masking effect was simply a consequence of acoustic amplification.

Furthermore, it is plausible that the reduction in tinnitus-related distress observed in our study may not solely stem from the masking effect of amplification. The improvement in hearing itself may reduce the overall hearing handicap, thereby decreasing listening effort and cognitive load, which in turn could lower stress levels and enhance the ability to tolerate tinnitus [34].

Our findings align with a substantial body of research emphasising the benefits of sound enrichment or sound therapy—including hearing aids, sound generators, and enriched acoustic environments—in alleviating tinnitus-related handicap. For instance, a randomized controlled trial found that hearing aid use combined with counselling led to significantly greater reductions in tinnitus severity compared to counselling alone [35]. Another study demonstrated that exposure to a personalised enriched acoustic environment for just one hour daily over four months resulted in clinically relevant decreases in THI scores [36]. Further, a large-scale study involving nearly 500 participants showed meaningful improvements in both loudness and annoyance VAS scores over 3 to 12 months of hearing aid-based sound therapy [37]. A broader systematic review of studies since 2011 found that hearing aids or combination devices reduced tinnitus distress in approximately 68% of cases [38]. Together, this evidence supports the concept that amplification contributes to tinnitus relief primarily through enriching the auditory environment, thereby decreasing tinnitus intrusiveness.

During hearing aid fitting phase, no formal assessment of tinnitus perception was conducted, as the target insertion gain has not yet been achieved. Patients were informed of this and were advised not to place undue importance on any perceived lack of benefit at that stage. Nonetheless, it is important to acknowledge that even at this early stage, the improved auditory perception afforded by the hearing aid fitting could potentially lead to a reduction in the subjective perception of tinnitus.

In addition to the masking effect, other mechanisms may contribute to the reduction in tinnitus intrusiveness following hearing aid use. These include improved audibility of environmental sounds, which may redirect attention away from tinnitus, and the restoration of auditory input in previously deprived regions of the cochlea. This stimulation could promote reorganisation of auditory pathways, potentially mitigating aberrant neural activity associated with tinnitus perception [34].

It is important to clarify that in our study the effectiveness of the hearing aid use for the tinnitus was assessed through the THI questionnaire, which primarily evaluates the reaction and handicap associated with tinnitus rather than its perceptual attributes, such as loudness or pitch. Perception and reaction are distinct constructs: while tinnitus perception refers to the auditory sensation itself, reaction reflects the emotional and functional impact that tinnitus has on the patient’s quality of life. Our findings therefore indicate a reduction in tinnitus-related handicap, rather than a direct modification of tinnitus perception.

When analysing the MCID, nearly all patients—except for six (three from each group)—achieved a minimal clinically important difference, showing an improvement in THI score greater than 11 points. Notably, as highlighted by the results, the group with ski-slope hearing loss demonstrated a greater reduction in THI scores, suggesting a more substantial perceived benefit. This clinically relevant finding may lay the groundwork for identifying different tinnitus pathophysiological mechanisms and phenotypes, potentially allowing clinicians to predict which patients, based on their hearing loss profile, are more likely to benefit from amplification. However, these are merely hypotheses that require confirmation through specifically designed studies.

While the precise impact of hearing aids features on tinnitus remains to be determined, our findings indicate that the level of tinnitus-related handicap, as assessed by the THI, was nonetheless reduced.

To date, only two non-randomised studies have assessed the effects of frequency lowering on tinnitus [17]. One study reported an 81% reduction in tinnitus, showing better outcomes compared to wide dynamic range compression alone. Conversely, the second study found opposite results, with tinnitus improvement in 44% of patients using wide dynamic range compression, compared to only 19% in those using frequency lowering [17]. Although our study is not a randomised clinical trial, it may contribute valuable insights to the existing scientific literature. Further studies are needed to determine whether frequency-lowering algorithms could play a role in tinnitus management.

In our correlation analysis, we investigated whether tinnitus level might correlate with tinnitus duration, hypothesizing that intensity could vary depending on the duration. However, no significant correlation was found. Similarly, we examined whether the MCID and the post-treatment THI scores were correlated with tinnitus duration or pitch; again, in line with other studies, we found no significant relationship between these variables [39,40]. The rationale for conducting these correlations lies in the hypothesis that, in individuals with tinnitus, neural reorganization might occur at various levels within the auditory pathway. A longer tinnitus duration may indeed establish a state of altered chronic neural response properties and reorganization within cortical tonotopic maps, potentially making tinnitus more persistent and resistant to treatment through auditory stimulation [4].

### Study Limitation and Future Perspectives

This study certainly has some limitations: (1) it is based on a small sample size. Conducting a prospective study with a larger sample size, possibly multicentric, could help confirm the findings of our work; (2) patients with ski-slope hearing loss may have areas of dead regions, which could affect the results. Evaluating this group of patients concerning the specific characteristics of their audiometric curves could be useful; (3) the sound processing strategies of hearing aids, the prescription methods used, and the characteristics of noise reduction systems or frequency lowering could influence the benefits obtained in our sample. It would be interesting to stratify patients using different digital processing systems to identify any potential differences.

It is important to acknowledge that in our study the primary reason for seeking clinical consultation was not systematically distinguished between hearing loss and tinnitus. Some patients may have presented primarily for hearing loss and only secondarily reported tinnitus, whereas others may have sought help predominantly for tinnitus. This distinction could be relevant, as the motivation for seeking amplification might influence the perception of benefit and the degree of improvement in THI scores. Unfortunately, our dataset does not allow us to stratify patients based on this variable, which represents a limitation of the present study and should be addressed in future research.

Our research could inspire future research, offering insights that can be explored and refined in subsequent studies. Given the initial findings, a larger-scale, prospective approach could further examine the efficacy of different hearing aid fittings in managing tinnitus across singular audiometric profiles. This is due to the increasing sophistication of modern signal processing algorithms, as well as the potential role that machine learning and artificial intelligence could play in future hearing aids. Such technologies might, for instance, analyse patients’ listening habits and exploit specific tinnitus characteristics to tailor amplification more effectively. Of course, these are merely speculative considerations proposed by us, the authors, but they represent possibilities that may become available in the not-too-distant future.

## 5. Conclusions

Although clear perceptual differences were observed between the groups in terms of tinnitus frequency, intensity, and edge frequency, both the high-frequency gently sloping and ski-slope hearing loss groups demonstrated statistically significant improvements in THI scores after six months of hearing aid use. Both the high-frequency gently sloping and ski-slope hearing loss groups demonstrated statistically significant improvements in THI scores after six months of hearing aid use. Since the THI assesses the reaction and handicap related to tinnitus, the obtained results specifically support the role of hearing aid amplification in reducing the burden associated with tinnitus, rather than its perception per se. The observed lack of significant correlation between tinnitus duration, level, pitch and post-treatment THI scores aligns with existing literature, while acoustic stimulation offers symptomatic relief. Future research should aim for larger, multicentric designs to validate these findings and refine therapeutic approaches tailored to distinct auditory profiles.

## Figures and Tables

**Table 1:** Study population.

	High-Frequency Gently Sloping			Ski-Slope		
	25th	Median	75th	25th	Median	75th
Patient’s age	58	70	77	60	69	75
Tinnitus Duration (years)	4	4	7	2	4	5

**Table 2:** Tinnitus characteristics and THI scores of the study population.

	High-Frequency Gently Sloping			Ski-Slope					
	25th	Median	75th	25th	Median	75th	Mann–Whitney U	Z	*p*-Value
Right tinnitus level (dB SL)	5	10	15	0	5	20	174	−0.193	0.847
Right tinnitus pitch (Hz)	1600	1700	3100	1200	1300	1600	91.5	−2.609	0.009 *
Left tinnitus level (dB SL)	10	15	15	0	10	15	143	−1.115	0.265
Left tinnitus pitch (Hz)	1500	1700	2000	1200	1400	1600	90.5	−2.665	0.008 *
Right edge frequency (Hz)	1500	2000	3000	1000	1000	1000	75.5	−3.205	0.001 *
Left edge frequency (Hz)	1500	1500	3000	1000	1000	1000	70.5	−3.341	0.001 *
Pre-treatment THI	24	32	44	46	62	68	92.5	−2.573	0.010 *
Post-treatment THI	8	12	18	8	18	30	116	−1.894	0.058

* statistically significant.

**Table 3:** THI pre- and post-treatment.

	Pre-Treatment THI			Post-Treatment THI				
	25th	Median	75th	25th	Median	75th	Z	*p*-Value
High-frequency gently sloping	24.00	32.00	44.00	8.00	12.00	18.00	−3.825	<0.001 *
Ski-slope	46.00	62.00	68.00	8.00	18.00	30.00	−3.825	<0.001 *

* statistically significant.

**Table 4:** Correlation analysis between tinnitus duration and tinnitus level.

		Right Tinnitus Level	Left Tinnitus Level
Correlation coefficient	Tinnitus duration	0.093	−0.177
*p*		0.578	0.286

**Table 5:** Correlation analysis between tinnitus duration and MCID.

		High-Frequency Gently Sloping	Ski-Slope
		Tinnitus duration (years)	
Correlation coefficient	MCID	−0.126	0.408
*p*		0.609	0.083

**Table 6:** Correlation analysis between tinnitus pitch and post-treatment THI.

		Right Tinnitus Pitch	Left Tinnitus Pitch
Correlation coefficient	Post-treatment THI	−0.096	−0.152
*p*		0.567	0.361

## Data Availability

The data presented in this study are available on request from the corresponding author due to the presence of information concerning the personal clinical data of the patients included in the study.

## Abbreviations

The following abbreviations are used in this manuscript:

CBP	Cognitive behavioural therapy
TRT	Tinnitus retraining therapy
PTA	Pure Tone Average
THI	Tinnitus Handicap Inventory
RIC	Receiver-In-Canal
MCID	Minimal clinically important difference
